# Reduction in DALYs lost due to soil-transmitted helminthiases and schistosomiasis from 2000 to 2019 is parallel to the increase in coverage of the global control programmes

**DOI:** 10.1371/journal.pntd.0010575

**Published:** 2022-07-07

**Authors:** Antonio Montresor, Pauline Mwinzi, Denise Mupfasoni, Amadou Garba

**Affiliations:** 1 Department of Control of Neglected Tropical Diseases, World Health Organization, Geneva, Switzerland; 2 Expanded Special Project for Elimination of Neglected Tropical Diseases, World Health Organization, Regional Office for Africa, Brazzaville, Congo; University of Florida, UNITED STATES

## Abstract

Preventive chemotherapy interventions for the control of soil-transmitted helminthiases (STH) and schistosomiasis scaled up from a global coverage level of around 5% in the year 2000 to a coverage that surpassed 60% in the year 2019. The present paper analyses the concomitant reduction in the number of disability-adjusted life years (DALYs) lost due to STH and schistosomiasis during the same period, from 6.3 to 3.5 million DALYs. The cumulative gain during the 19-year period was estimated at over 26 million DALYs. Given the low cost of the intervention, our study suggests that deworming for STH and schistosomiasis is one of the most cost-effective public health interventions.

## Author summary

Schistosomiasis and intestinal parasites cause significant morbidity and, in endemic countries, WHO recommends the implementation of periodical large scale distribution of anthelminthic drugs (deworming) for the control of the morbidity due to these infections.

Deworming significantly scaled up from the year 2000 to 2019 reaching the majority of children at risk, as a consequence the number of infected individuals and the severity of the infections has been significantly reduced. This is reflected on the significant reduction of number of DALYs lost in the last two decades for these two infections.

## Background

Soil-transmitted helminthiases (STH) are a group of infections caused by four parasite species: *Ascaris lumbricoides*, *Trichuris trichiura*, *Ancylostoma duodenale* and *Necator americanus*. The two latter species are indistinguishable by microscopy and are normally reported together as “hookworms”. Schistosomiasis is caused mainly by three schistosome species: *Schistosoma haematobium*, *S*. *mansoni* and *S*. *japonicum*. These two groups of parasites are transmitted mainly in tropical countries where sanitation is poor, causing significant morbidity including nutritional disturbance and, in cases of human schistosomiasis, granuloma, organ pathology and sometimes cancer [1].

The morbidity caused by STH and schistosomiasis is proportional to the number of worms infecting the host [1]. Infections of moderate to heavy intensity are responsible for the greater part of the morbidity attributable to these two groups of parasites [2].

In countries where STH and schistosomiasis are endemic, in addition to efforts to improve sanitation and behavioural change interventions, the World Health Organization (WHO) recommends preventive chemotherapy (PC), a periodic treatment with anthelminthic medicines, to different groups of populations at risk [2].

Before 2000, few countries were implementing PC interventions at national level. Then, thanks to the progressive engagement of decision-makers in endemic countries, partners and the availability of three medicine donations managed by WHO—albendazole (from Johnson & Johnson), mebendazole (from GlaxoSmithKline) and praziquantel (from Merck) [3]—PC coverage scaled up progressively covering the majority of children in endemic areas. As a consequence of these large-scale interventions, the morbidity caused by STH and schistosomiasis has been progressively reduced.

The aim of this study is to evaluate if the scaling up of PC interventions between 2000 and 2019 is associated with the parallel decline in the morbidity caused by STH and schistosomiasis and provide some recommendations on next steps.

## Material and method

### Estimation of the morbidity caused by STH and schistosomiasis, 2000–2019

The disability-adjusted life year (DALY) is an internationally accepted indicator for the estimation of the burden of disease; this measure includes information on both mortality and morbidity [4]. WHO publishes, at regular intervals in the Global Health Estimates, the estimated number of DALYs lost for a large number of diseases or conditions (including DALYs lost due to STH and schistosomiasis) [5]. For this study we considered the estimation for DALYs lost due to STH and schistosomiasis in 2000 (before PC activities were scaled up), in 2010 (when PC programmes were significantly expanded) and in 2019 (the year for which the latest DALY estimation is available).

### Global coverage of PC, 2000–2019

WHO manages three medicine donations for the control of STH and schistosomiasis. Every year, countries in need of anthelminthics for this purpose submit requests to WHO indicating the number of anthelminthic tablets they plan to distribute over the upcoming year. Countries submit a report on the coverage results from the previous year. In this way WHO manages the donations and documents the global progress of PC coverage [6–7]. For consistency with the morbidity data, in this study we considered the PC coverage data at three time points: 2000, 2010 and 2019. We also estimated the cumulative amount of donated medicines at the three time points.

## Results

### Decrease in DALYs lost due to STH, 2000–2019

**Fig 1:**
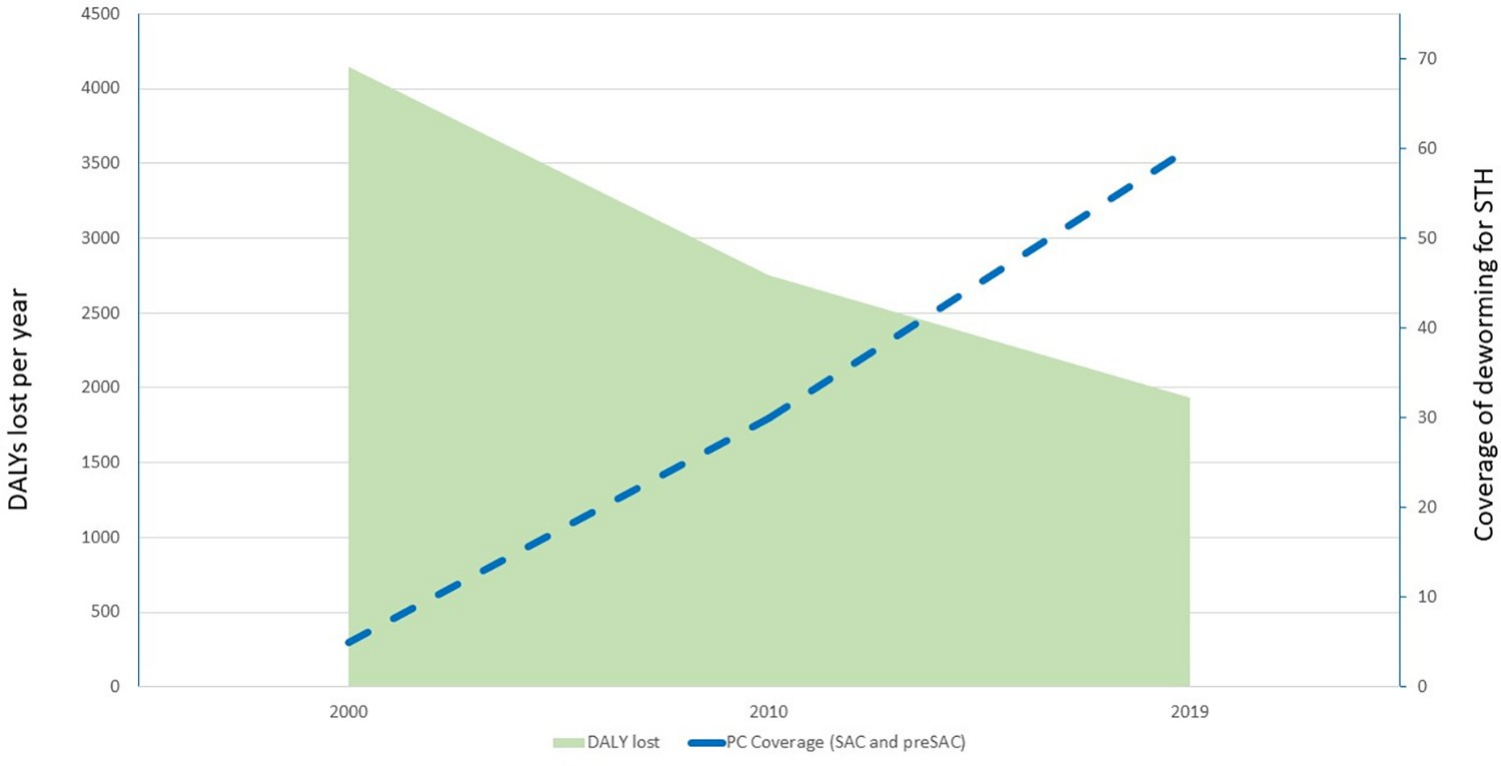
Annual number of DALYs lost due to STH 2000–2019 and coverage of preventive chemotherapy programmes for the control of STH in children.

The number of DALYs lost due to STH was estimated at over 4 million in 2000, decreasing to 2.7 million in 2010 and to an estimated 1.9 million in 2019 (53% reduction from 2000). **Fig 1** presents this decrease in parallel with the scaling up of the global coverage of the PC programme. If we observe the impact of the control intervention by parasite species (**Table 1**), we note that the reduction in absolute number has been more relevant for hookworm infections (from 2 million DALYs in 2000 to 0.7 million DALYs in 2019), while proportionally the decrease in DALYs has been more pronounced for *T*. *trichiura* infections (in 2019 over 56% DALYs reduction compared with the morbidity caused in 2000). If we analyse the changes in DALYs lost in the different age groups (**Table 2**), we note that the reduction has been higher in school-aged children (59%) and preschool-aged children (50%), but that the benefits have also been extended to other age groups.

**Table 1:** Reduction in DALYs lost due to STH and schistosomiasis, 2000–2020, by species.

	DALYs lost		
Disease	2000	2010 (reduction from 2000)	2019 (reduction from 2000)
Ascariasis	1604	972 (39%)	749 (53.3%)
Trichuriasis	532	272 (48%)	232 (56.4%)
Hookworm infection	2099	1511 (24%)	962 (54.1%)
**Total STH**	**4235**	**2755** **(36%)**	**1933** **(54.1%)**
**Total schistosomiasis**	**2220**	**2100** **(4.5%)**	**1628** **(24.2%)**
**Total STH + schistosomiasis**	6365	4855 (23.7%)	3615 (43.2%)

**Table 2:** Reduction in DALYs lost due to STH and schistosomiasis, 2000–2020 by age group.

	2000				2019 (reduction from 2000)			
	1–59 months	5–14 years	15–29 years	> 30 years	1–59 months	5–14 years	15–29 years	> 30 years
Ascariasis	337 709	631 059	310 883	324 270	179 021 (46.99%)	248 416 (60.64%)	143 250 (53.92%)	177 932 (43.13%)
Trichuriasis	29 728	170 295	214 103	118 059	13 221 (55.53%)	59 604 (65.00%)	97 893 (54.28%)	61 225 (48.14%)
Hookworm infection	166 526	651 651	799 017	482 008	71 853 (56.85%)	284 355 (56.36%)	357 715 (55.23%)	248 531 (48.44%)
**Total STH**					264 095 (50.00%)	592 375 (59.23%)	598 858 (54.77%)	487 688 (47.24%)
Schistosomiasis	41 961	376 393	666 750	1 134 440	30 572 (27.14%)	259 491 (31.16%)	506 203 (24.08%)	831 578 (26.70%)

### Decrease in DALYs lost due to schistosomiasis, 2000–2019

**Fig 2:**
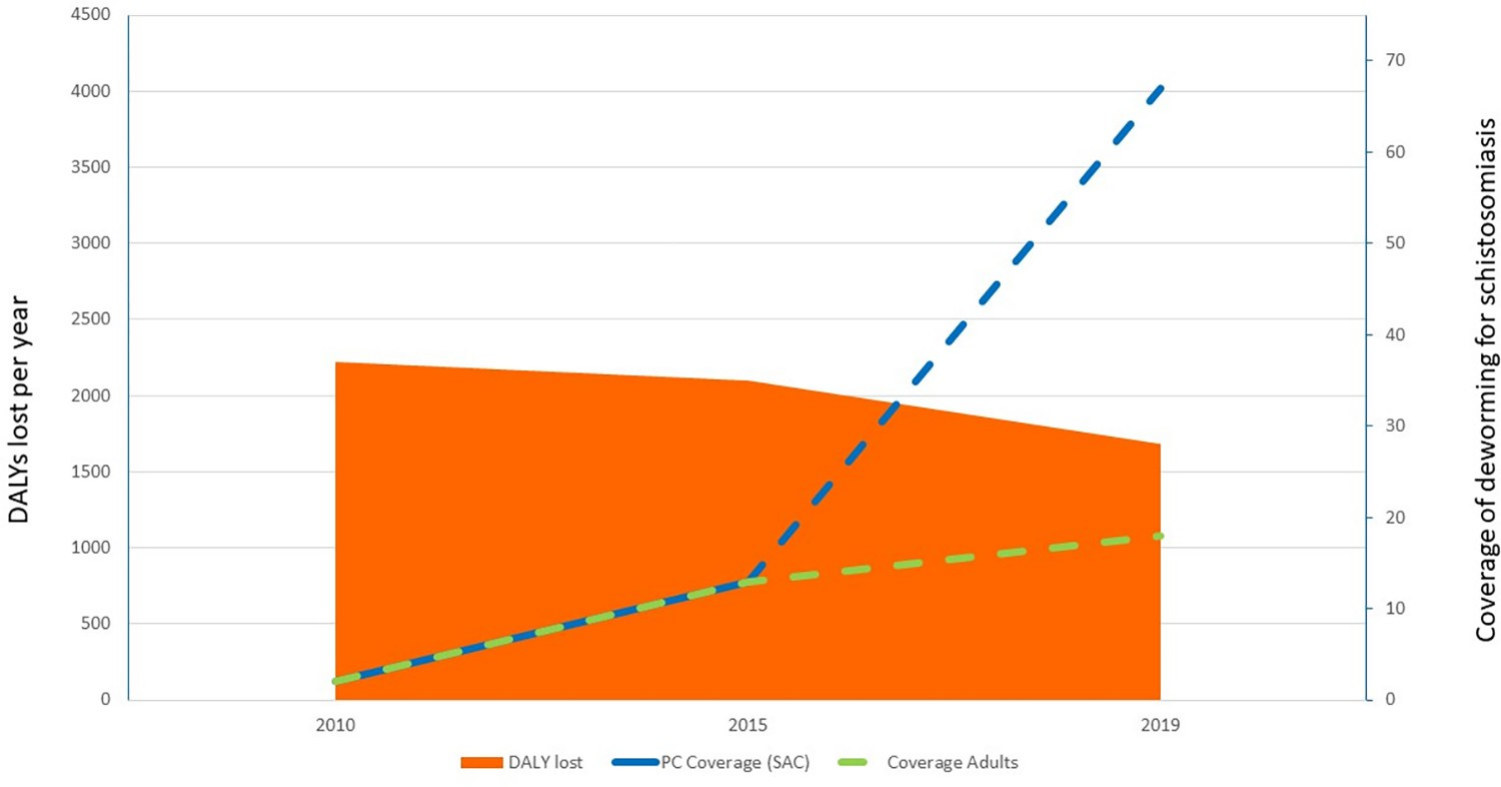
Annual number of DALYs lost for schistosomiasis 2000–2019 and coverage of preventive chemotherapy programmes for the control of schistosomiasis in children.

The number of DALYs lost due to schistosomiasis was estimated at over 2.2 million in the year 2000, decreasing to 2.1 million in 2010 and to an estimated 1.6 million in 2019 (24% reduction from 2000). **Fig 2** presents this decrease in parallel with the scaling up of the global coverage of the control programme. If we analyse the changes in DALYs lost in the different age groups (**Table 2**), we note that the proportion of reduction has been very little in preschool-aged children. This reflects the fact that they were not targeted between 2010 and 2019 due to the lack of approved anti-schistosomiasis treatment for this age group; it was of 31% in school-aged children and the benefit has also been extended to other age groups.

### Donated medicines for the control of STH and schistosomiasis

**Table 3** presents the cumulative quantities of donated anthelminthics for the control of STH and schistosomiasis. The Johnson & Johnson donation of mebendazole for STH started in 2007, with approximately 95 million tablets (corresponding to 95 million doses) donated until 2010 and an additional 1.2 billion doses donated in 2010–2019. The GlaxoSmithKline donation of albendazole for STH started in 2011, with more than 1.6 billion doses donated in 2011–2019. The Merck donation of praziquantel started in 2012, with 1 billion tablets donated in 2012–2019, corresponding to approximately 400 million doses (it is estimated that a child needs on average 2.5 tablets [8]).

**Table 3:** Cumulative amount of donated medicines for the control of STH at three time points.

Cumulative amount of donated tablets (in millions)			
	2000	2010	2019
Albendazole (for STH)	0	0	1666*
Mebendazole (for STH)	0	95*	1207*
STH programme global coverage	5%	30%	62%
Praziquantel (for schistosomiasis)	0	0	1000°
Schistosomiasis programme global coverage	3%	15%	68%

* For albendazole and mebendazole the number of tablets corresponds to the number of treatments.

° For praziquantel 1000 million corresponds to 400 million treatments.

### Coverage of PC for the control of STH, 2000–2019

In 2000, the coverage of PC interventions for the control of STH was less than 5% (with a few million children treated in a few pilot programmes). Since then, PC programmes for the control of STH have expanded significantly, reaching in 2010 over 275 million children (30% of the global child population at risk) and, in 2019, over 613 million children (60% coverage) [6,7] (**Fig 1**).

### Coverage of PC for the control of schistosomiasis, 2000–2019

Similarly to STH, with the exception of China and Egypt [7], only a few large-scale pilot programmes were in place for the control of schistosomiasis before 2000. In 2010, PC coverage was around 8% and in 2019 was 67% for children and 17% for adults (**Fig 2**).

## Discussion

The main limitation of the study is the fact that the limited number of datapoints (consequent to the fact that the Global Health Estimates are collected at time intervals of 5–10 years) did not allow us to evaluate statistically (i.e. by the calculation of the Pearson coefficient) the negative correlation between scaling up of coverage of deworming programmes and scaling down of the number of DALYs lost due to schistosomiasis and STH. However, we consider that both Figs 1 and 2 graphically show the association of the two events. Another possible limitation is the fact that the quality of DALYs estimates could have improved over the period 2000–2019 and therefore the decrease in the number of DALYs lost due to schistosomiasis and STH reflects these improved estimations rather than the effect of the control activities. Another possible limitation is that the possible contributors to the decrease in the number of DALYs lost during the period considered could have been due to other factors such as the general improvement of the sanitation services, accompanied by changes in behaviour consequent to health education activities. However, the UN-Water Global Analysis and Assessment of Sanitation and Drinking-Water (GLAAS) Report 2019 [9] estimates that, despite the availability of policies to improve the sanitation level in over 94% of countries, less than 15% of the countries have funds or human resources to implement their plans and that in 2019 more than 670 million people still lack basic sanitation.

In the large majority of cases, the presence of schistosomiasis in an area is indicative of the presence of STH [10]. For this reason, the distribution of praziquantel for the control of schistosomiasis is associated almost constantly with the distribution of albendazole or mebendazole for the control of STH. We can therefore consider the two programmes as completely integrated even if, when preschool-aged children are treated for STH, they cannot be treated for schistosomiasis due to the lack of approved anti-schistosomiasis treatment for this age group. As such, the number of DALYs averted with this integrated intervention between 2000 and 2019 is impressive: the number of DALYs lost due to both diseases fell from 6.4 million/year to 3.6 million/year, with a cumulative gain during the 19-year period of over 26 million DALYs.

It is remarkable that the reduction in DALYs lost for both STH and schistosomiasis in 2000–2019 was obtained not only in the group targeted by the PC intervention but also in other age groups. In our opinion this is because of several reasons: first, children comprise a large fraction of those infected second, the age groups are not fixed categories; every year new individuals enter the group and older ones move to the following group. Additionally, since children are recognized as the more active group in terms of open defecation [11], removing a large part of the worm burden in this group has also positive effects in reducing environmental contamination.

At this point it is important to stress that a relaxation of the control measures would diminish the benefits gained until now. The control measures should be adapted to the changes in epidemiology of the two infections. The frequency of PC should be reduced where the intervention obtained substantial results and the control intervention intensified in areas where the prevalence of both diseases is still high, according to the decision tree developed by WHO [1]. To adapt the PC frequency, endemic countries should conduct surveys to evaluate the impact of the control intervention. WHO is working on the development of a survey design that will enable a reduction in the sample size and therefore the cost of such surveys [12]. It is expected that this adaptation will result in a decrease of the need for anthelminthics [13] and facilitate endemic countries in supporting control programmes with local resources. In addition, a reduction of the need for medicines will also reduce the risk of development of drug resistance by the parasites.

The reduction in morbidity has been less relevant for schistosomiasis, since the donation of praziquantel started 5 years later than that for mebendazole. In addition, preschool-aged children are currently excluded by the PC intervention because of safety considerations. However, this age group suffers from schistosomiasis morbidity and is in need of treatment [14]; a praziquantel formulation adapted to this age group is under development [15]. The inclusion of preschool-aged children in PC for schistosomiasis and of women of reproductive age in PC for STH would be essential to expand the impact of PC in the next years. WHO recently developed a policy paper to support the scaling up of deworming of women of reproductive age [16].

Our study confirms the previous estimation of the reduction of the global burden due to STH [17] and documents the progress of the control of STH and schistosomiasis towards the elimination of morbidity as articulated by the WHO road map 2021–2030 [18–20].

The study also suggests that given the low cost for each individual treated, PC interventions for the control of STH and schistosomiasis [21] are one of the most cost-effective public health interventions [22].

Disclaimer: The authors are staff members of the World Health Organization. The authors alone are responsible for the views expressed in this article and they do not necessarily represent the decisions, policy or views of the World Health Organization.
